# Supplementary material for: Genetic drivers of congenital cardiac fibrosis
Source: Commun Biol. 2026 May 27;9:722. doi: 10.1038/s42003-026-10353-2 (PMC13216529; doi:10.1038/s42003-026-10353-2)
Supplement: Supplementary file 1 — Description of Additional Supplementary Materials [file 42003_2026_10353_MOESM1_ESM.pdf]

# Description of Additional Supplementary Files

**File name:** Supplementary Data 1

**Description:** Cardiac fibroblast markers

**File name:** Supplementary Data 2

**Description:** Genes associated with CHD

**File name:** Supplementary Data 3

**Description:** Genes with potential causal roles in cardiac fibrosis
